# Genomic predictions under different genetic architectures are impacted by mating designs

**DOI:** 10.1016/j.vas.2024.100373

**Published:** 2024-06-19

**Authors:** Sahar Ansari, Navid Ghavi Hossein-Zadeh, Abdol Ahad Shadparvar

**Affiliations:** Department of Animal Science, Faculty of Agricultural Sciences, University of Guilan, Rasht, 41635-1314, Iran

**Keywords:** Assortative mating, Genomic breeding value, Genomic prediction accuracy, Genomic selection, Prediction bias

## Abstract

Mating in animal communities must be managed in a way that assures the performance increase in the progenies without increasing the rate of inbreeding. It has currently become possible to identify millions of single nucleotide polymorphisms (SNPs), and it is feasible to select animals based on genome-wide marker profiles. This study aimed to evaluate the impact of five mating designs among individuals (random, positive and negative assortative, minimized and maximized inbreeding) on genomic prediction accuracy. The choice of these five particular mating designs provides a thorough analysis of the way genetic diversity, relatedness, inbreeding, and biological conditions influence the accuracy of genomic predictions. Utilizing a stochastic simulation technique, various marker and quantitative trait loci (QTL) densities were taken into account. The heritabilities of a simulated trait were 0.05, 0.30, and 0.60. A validation population that only had genotypic records was taken into consideration, and a reference population that had both genotypic and phenotypic records was considered for every simulation scenario. By measuring the correlation between estimated and true breeding values, the prediction accuracy was calculated. Computing the regression of true genomic breeding value on estimated genomic breeding value allowed for the examination of prediction bias. The scenario with a positive assortative mating design had the highest accuracy of genomic prediction (0.733 ± 0.003 to 0.966 ± 0.001). In a case of negative assortative mating, the genomic evaluation's accuracy was lowest (0.680 ± 0.011 to 0.899 ± 0.003). Applying the positive assortative mating design resulted in the unbiased regression coefficients of true genomic breeding value on estimated genomic breeding value. Based on the current results, it is suggested to implement positive assortative mating in genomic evaluation programs to obtain unbiased genomic predictions with greater accuracy. This study implies that animal breeding programs can improve offspring performance without compromising genetic health by carefully managing mating strategies based on genetic diversity, relatedness, and inbreeding levels. To maximize breeding results and ensure long-term genetic improvement in animal populations, this study highlights the importance of considering different mating designs when evaluating genomic information. When incorporating positive assortative mating or other mating schemes into genomic evaluation programs, it is critical to consider the complex relationship between gene interactions, environmental influences, and genetic drift to ensure the stability and effectiveness of breeding efforts. Further research and comprehensive analyzes are needed to fully understand the impact of these factors and their possible complex interactions on the accuracy of genomic prediction and to develop strategies that optimize breeding outcomes in animal populations.

## 1 Introduction

During recent decades, along with the progress in molecular genetics, it has been possible to detect numerous markers in a small region of the genome. The main features of the applied markers for determining QTL are the existence of more than one allele in the population, accessibility to the entire genome, and simple and cost-effective experimental methods (Mukhopadhyay et al., 2020). Using the marker information in animal breeding programs, the marker-assisted selection (MAS) theory has been developed extensively (Mukhopadhyay et al., 2020). It is possible to estimate the genetic value of an individual using the markers linked with QTL. This genetic value may be more accurate than the genetic value calculated using pedigree and phenotypic information. Despite the utility of MAS demonstrated in research studies, its use is limited, and consequently genetic progress leveraging MAS has not been considerable. In a MAS program, the markers used for selection are evaluated thoroughly via robust association analyses with relatively high thresholds for attaining statistical significance (Weigel, 2017). The majority of QTL affecting traits would be lost through this assessment, especially those that have small effects (Ibtisham et al., 2017; Weigel, 2017). In conclusion, only a small percentage of the genetic variance for traits that are controlled by numerous loci with small effects can be attributed to the markers (Hayes et al. 2009). In recent years, as the cost of high throughput sequencing has declined, it has become possible to identify millions of SNPs, and it is currently feasible to select animals based on genome-wide marker profiles (Khanzadeh et al., 2020).

MAS is helpful for traits that have not benefitted from current evaluation methods because MAS uses phenotypic and molecular marker information simultaneously (Ibtisham et al., 2017; Mukhopadhyay et al., 2020). Advances in genomic selection have been associated with the detection of multiple SNPs through genome sequencing (Hayes et al., 2009). The process of evaluating and choosing potential candidates by applying genomic information is known as genomic selection. It is assumed that the marker is in the linkage disequilibrium phase with QTL and that the method's key feature is to cover the entire genome with dense markers, so that these markers explain all genetic variance (Goddard & Hayes, 2007; Johnsson, 2023). Marker effects are estimated based on genotype and phenotype information, and based on these estimated marker effects, the genomic breeding values are predicted (Mueller & Van Eenennaam, 2022). The need for a large number of markers and genotypic costs were two main limitations for the implementation of genomic selection. These limitations have been overcome in most animal species after genome sequencing of domestic animals and the identification of many SNP due to significant developments in genotyping technologies (Hayes et al., 2009; Ibañez-Escriche & Gonzalez-Recio, 2011).

A mating design can be considered as a group of regulations to select appropriate mating strategies. In random mating, mates are selected randomly (Bourdon, 2014). No phenotypic records or genetic predictions are needed by random mating, and making mating decisions requires little time. Therefore, when phenotypic information is inaccessible or where there are numerous animals that other mating methods are inappropriate, random mating is applicable in commercial breeding plans (Bourdon, 2014). The two types of assortative mating are negative assortative mating (mating of dissimilar individuals) and positive assortative mating (mating of similar individuals). “Similar” in this situation conventionally signifies having identical performance or genetic prediction for a trait or complex of traits (Bourdon, 2014). Any mating design that is not random is certainly a type of assortative mating. For conducting assortative mating, it is necessary to have phenotypic records, genetic values, or some other mating indicators (Bourdon, 2014). Positive assortative mating generates more genetic and phenotypic variation in the progenies than would be expected in an equivalent randomly mated population. On the other hand, negative assortative mating inclines to reduce variation. Negative assortative mating tends to generate more intermediate forms and decrease the number of extreme progenies (Bourdon, 2014). This mating design is not an appropriate strategy to accelerate the directional genetic progress rate. Decreased genetic variation reduces selection response. However, if the principal objective is to extend phenotypic uniformity about some intermediate optimum, negative assortative mating can be advantageous (Bourdon, 2014).

Assortative mating is characterized by numerous non-random mating patterns (Jiang et al., 2013). Many key evolutionary outcomes result from assortative mating. As a result, the variance of quantitative traits rises. Positive assortative mating enhances homozygosity within loci and increases linkage disequilibrium (LD) between loci (Lynch & Walsh, 1998). Deviations from the Hardy-Weinberg equilibrium due to such mating patterns can introduce biases in association mapping studies and quantitative genetic parameter estimates (Gimelfarb, 1986; Redden & Allison, 2006). Premating isolation between populations with divergent phenotypes is facilitated by assortative mating, which is another important factor in speciation (Coyne & Orr, 2004; Bolnick & Kirkpatrick, 2012). Through an increase in LD, assortative mating has been proposed to improve estimates of additive genetic variance and heritability (Hedrick, 2017). Theoretical analyses have shown that assortative mating can result in LD, which in turn can affect estimates of additive genetic variance and heritability (Wright, 1921; Crow & Felsenstein, 1968). Moreover, the frequency of homozygotes would be increased by inbreeding and assortative mating when compared to random mating (Hedrick, 2017). It is suggested that when assortative mating occurs at a high enough frequency to affect LD, it may also affect unlinked or loose loci because the amount of LD generated is independent of the rate of recombination among loci. Additionally, it was predicted by Wright (1921) and Crow & Felsenstein (1968) that assortative mating would have a significant impact on heritability and additive genetic variance if multiple loci were involved, likely due to the cumulative impact of LD between these many loci (Hedrick, 2017). Tsuruta et al. (2021) stated that a change in heritability, due to selection, shows the impact that the Bulmer effect has on the decrease in between-family variation, whereas assortative mating affects the Mendelian sampling variation or within-family variance. Also, they reported that though directional selection can decline heritability, positive assortative mating, which was greatly associated with considerable genetic progress, could lessen the reduction in heritability for a trait under strong selection and could impact bias in genomic predictions (Tsuruta et al., 2021). Zhang et al. (2022), in a simulation study, observed that in order to ensure a specific genetic variation, genomic mating designs—maximum genetic gain, minimum inbreeding, and maximum variance between families—cannot only yield greater genetic gains than positive assortative mating but also successfully reduce inbreeding rates and sluggish the rate at which genetic variation is lost. Gowane et al. (2018) stated that the variance among offspring would be enhanced by assortative mating and higher correlations between estimated and true breeding values could be observed.

Matings in animal populations must be managed in a way that assures the performance increase in the progenies and also does not lead to an increase in the inbreeding rate. Inbreeding is one of the chief factors that must be controlled in animal breeding plans to keep the reproduction potential and survival rate of animals (Eteqadi et al., 2015; Hashemi & Ghavi Hossein-Zadeh, 2020). To prevent inbreeding depression or the depletion of additive genetic variance, a breeding program for high-performance livestock breeds aims to maximize genetic gain while maintaining an adequate effective size of the breed. Limiting the rate of increase in mean kinship makes it possible to maintain an adequate effective size. The goal of this optimization problem is to maximize the mean breeding value in the offspring while limiting the increase in mean kinship in the population. As a result, the optimal contributions of the candidates for selection are found through these means. This method is the traditional method of optimum contribution selection (OCS) (Wellman, 2019). OCS is a selection strategy that works well for striking a balance between genetic gain and inbreeding rate (van der Werf, 2022). By limiting the relatedness of offspring, this selection process limits the rate of inbreeding while optimizing genetic gain in the following generation. The degree of kinship among offspring and the rate of inbreeding in subsequent generations can both be effectively regulated by OCS, which ultimately preserves genetic diversity (Wang et al., 2017). By using the stochastic simulation method, this study sought to evaluate the impact of five different mating schemes (random, positive and negative assortative mating, minimized and maximized inbreeding) under various genetic architectures (different numbers of markers and QTL, distinct heritability levels) on the accuracy of genomic predictions.

## 2 Materials and methods

### 2.1 Simulation

Based on a forward-in-time process, the QMSim software (Sargolzaei & Schenkel, 2009) was utilized to simulate the populations. A base population, also referred to as the historical population, consisting of 1000 unrelated animals (500 males and 500 females) was simulated to achieve a mutation-drift equilibrium. Within this population, gametes from the male and female gametic pools randomly unite to produce offspring. Its size increased by 1000 generations after that, reaching 10,000. Until generation 2020, the population continuously decreased from 10,000 to 4,000 individuals, creating LD. 500 males and 3,500 females who were chosen at random from members of the previous historical generation were mated for ten more generations to develop the population. This was done under the assumption that each dam would have five offspring and that the number of dams would increase exponentially. From the last generation of the expanded population, 50 males and 3500 females were mated to produce another 10 generations in order to start a new population. The training set was made up of individuals from the third through ninth generations, while everyone from the tenth generation made up the validation set. In the new population, five mating designs were considered as follows: random, positive and negative assortative, and minimized and maximized inbreeding. The mating design that maximizes inbreeding permits the quick production of an inbred line (Sargolzaei & Schenkel, 2009). The simulated Annealing method is used to optimize inbreeding (Sonesson & Meuwissen, 2000). The simulated annealing technique modifies the Metropolis-Hastings algorithm for the global optimization problem. According to Sargolzaei & Schenkel (2009), the temperature was first adjusted to 0.5 and subsequently lowered by a factor of 0.9. Random numbers are generated using the Mersenne Twister algorithm, a high quality, fast random number generator (Sargolzaei & Schenkel, 2009). Sires and dams mate in the assortative mating design according to how similar (positive) or different (negative) the estimated breeding values are.

One chromosome, measuring 100 cM in length, made up the simulated genome. 350, 650, and 950 bi-allelic markers distributed equally throughout the genome were considered to examine the effect of SNP density on the accuracy of genomic prediction. Among the markers, 50, 150, and 200 QTLs were assigned at random. From a gamma distribution with a shape parameter of 0.4, QTL allelic effects were sampled. It was assumed that the markers and QTLs would undergo mutation at a rate of 2.5 × 10^-5^ per locus per generation (Solberg et al. 2008). A simulated trait had a phenotypic variance of 1.0 and heritabilities of 0.05, 0.30, and 0.60. Assuming only additive QTL effects, each individual's true breeding value (TBV) was equal to the sum of the QTL allele substitution effects. Addition of residuals to TBVs, randomly selected from a normal distribution with a mean of zero, resulted in the production of phenotypes. Twenty replicates were simulated for each scenario. The accuracy of the genomic estimated breeding value (GEBV) prediction was assessed using the correlation between the true breeding value (BV) and the genomic predicted BV (r_TBV,GEBV_).

### 2.2 Calculation of linkage disequilibrium

The measurement of linkage disequilibrium (LD) was done using the square of the correlation between the alleles at two loci, or r^2^ (Hill & Robertson, 1968):

$$r^{2}=\frac{D^{2}}{f\left(A\right)f\left(a\right)f\left(B\right)f\left(b\right)}$$

where D = f(AB) − f(A) f(B). Haplotype AB and alleles A, a, B, and b are represented by the observed frequencies f(AB), f(A), f(a), f(B), and f(b), respectively. In the current study, when the number of markers is equal to 650 and a positive assortative mating design is used, linkage disequilibrium between SNP pairs was calculated based on different distances between SNPs in each generation of the reference population. Also, average linkage disequilibrium was calculated for different heritability levels and QTL number, when the number of markers is equal to 650 and a positive assortative mating design was implemented.

### 2.3 Marker effects prediction

To estimate marker effects, two methods were used: the Bayesian generalized linear regression (BGLR) package of the R program (De los Campos & Rodriguez, 2014) and the ridge regression best linear unbiased prediction (RR-BLUP) model (Hoerl & Kennard 1970):

y=1μ+Xg+e

where **y** is the vector of phenotypic values, **μ** is the overall mean, **X** is the matrix of marker genotypes for each animal (the SNP genotypes were coded as the number of copies of one SNP allele, i.e., 0, 1, or 2), **g** represents the vector of marker effects, and **e** is a vector of random error with a distribution of N(0,$\sigma_{e}^{2}$). $\sigma_{e}^{2}\;$is the residual variance. To obtain breeding values, the mixed model equation is:

$$\left[\begin{array}{cc} {1^{\prime }}_{n}1_{n} & {1^{\prime }}_{n}X\\ X^{\prime }1_{n} & X^{\prime }X+I\lambda \end{array}\right]\;\left[\begin{array}{c} \mu^{\prime }\\ g^{\prime } \end{array}\right]=\left[\begin{array}{c} {1^{\prime }}_{n}y\\ X^{\prime }y \end{array}\right]$$

where **X** represents the marker genotype matrix for every animal, and λ stands for the ridge regression factor =$\frac{\sigma_{e}^{2}}{\sigma_{g}^{2}}$, $\sigma_{g}^{2}$ is a common variance for each marker effect and **I** is an identity matrix. This is a counterpart to VanRaden's GBLUP method (VanRaden, 2008).

The GEBV of the animals in the validation set was computed as follows:

$$GEBV=X\hat{g}$$

where **X** represents the marker genotype matrix for every animal, while ĝ represents the estimated marker effects vector. By calculating the regression of true genomic breeding value on estimated genomic breeding value, the value of prediction bias can be investigated. For this regression to be considered unbiased, its expected value must equal one. A SAS program's simple linear regression procedure (PROC REG) was used to estimate the regression coefficients of true genomic breeding value on estimated genomic breeding value.

## 3 Results

### 3.1 Accuracy of genomic prediction

**Fig. 1:**
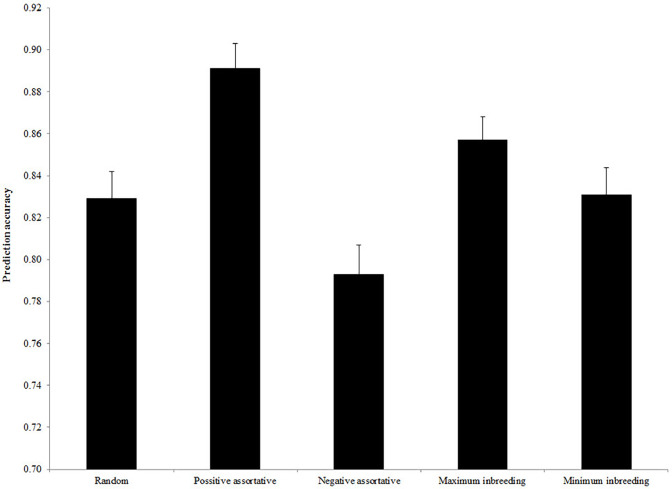
The mean estimated accuracy of genomic predictions (±SE) for different mating designs.

**Fig. 2:**
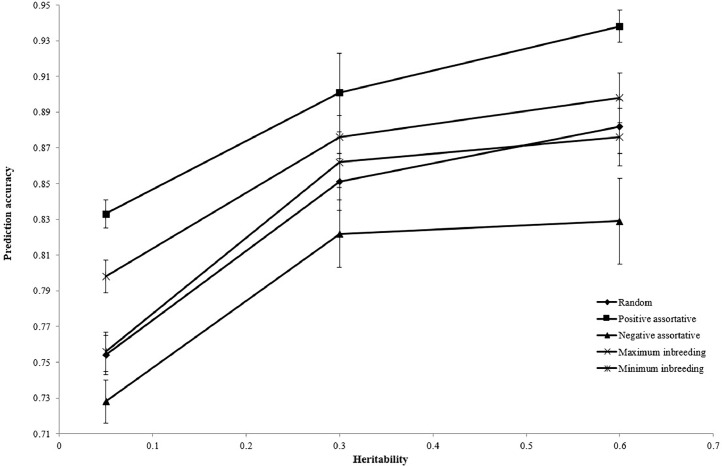
The mean estimated accuracy of genomic predictions (±SE) for different levels of heritability.

**Fig. 3:**
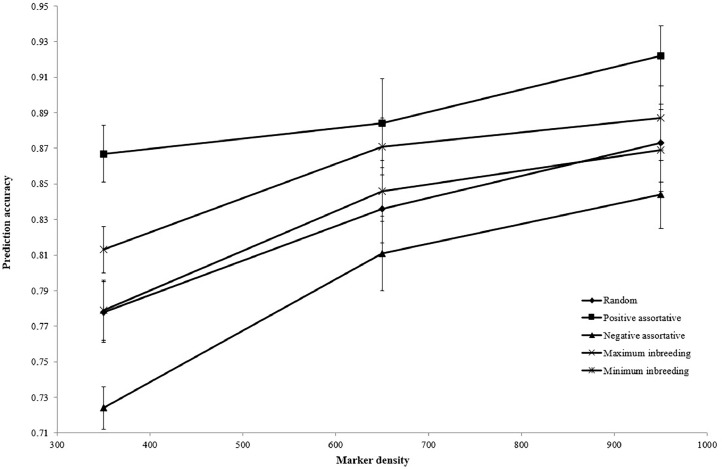
The mean estimated accuracy of genomic predictions (±SE) for different marker densities.

**Fig. 4:**
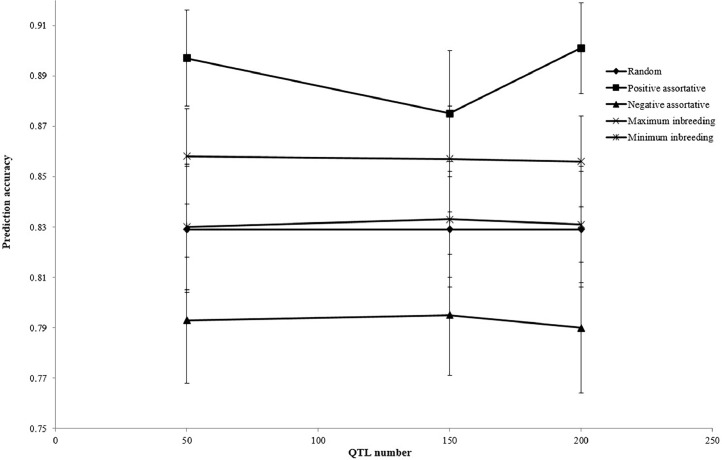
The mean estimated accuracy of genomic predictions (±SE) for different QTL numbers.

The estimated accuracy of genomic prediction for different mating designs is presented in Fig. 1. Implementation of positive assortative mating provided the greatest prediction accuracy (0.891), but negative assortative mating had the least accuracy (0.793). Fig. 2, Fig. 3, Fig. 4 illustrate the estimated accuracy of genomic prediction at various heritability, marker density, and QTL numbers. For all mating designs, an increase in heritability level increased the accuracy of genomic prediction. When marker density changed from 350 to 950, genomic accuracies increased for all mating designs. The genomic accuracy also showed no obvious variation while the QTL number increased from 50 to 200. Considering different genetic architectures of the trait, the positive assortative mating design provided the greatest accuracies, but the lowest genomic accuracies were observed when the negative assortative mating design was implemented (Fig. 1, Fig. 2, Fig. 3).

Estimated accuracies of genomic prediction and regression coefficient of true genomic breeding value on the estimated genomic breeding value in the reference and validation populations at different levels of heritability, marker density, and QTL number when random, positive assortative mating, negative assortative mating, minimized and maximized mating designs are depicted in Table 1, Table 2, Table 3, Table 4, Table 5, respectively. Comparing the accuracy of reference and validation populations in all scenarios showed a slight difference in accuracy. The implementation of positive assortative mating resulted in increased genomic accuracy for reference and validation populations across all heritability, QTL number, and marker density levels. If the true genomic breeding value's regression coefficients on the estimated genomic breeding values in the validation and reference populations approached unity, the estimated genomic breeding values would be unbiased. The results shown in Table 2 showed the unbiased regression coefficients of true genomic breeding value on the estimated genomic breeding value in the reference and validation populations when positive assortative mating was used. As a result, alternative mating strategies produced skewed estimates of genomic breeding values in addition to decreasing prediction accuracy. In general, higher heritability estimates for a given QTL number or marker density in a given mating design provided unbiased regression coefficients of true genomic breeding value on estimated genomic breeding value compared to low heritability estimates.

**Table 1:** Estimated accuracy of genomic prediction (SE) and regression coefficient of true genomic breeding value on the estimated genomic breeding value (b) in the reference (A_T_) and validation (A_V_) populations at different levels of heritability, marker density, and QTL number when the random mating design was implemented.

Marker no.	QTL no.	Heritability	A_T_(SE)	A_V_(SE)	b(SE)
350	50	0.05	0.712(0.006)	0.703 (0.008)	0.942(0.007)
		0.3	0.802(0.007)	0.794(0.007)	0.953 (0.009)
		0.6	0.825(0.003)	0.822(0.005)	1.001(0.008)
	150	0.05	0.721(0.005)	0.711(0.009)	0.948(0.02)
		0.3	0.804(0.005)	0.807(0.007)	0.991(0.007)
		0.6	0.827(0.004)	0.825(0.006)	1.002(0.007)
	200	0.05	0.714(0.007)	0.717(0.009)	0.959(0.01)
		0.3	0.806(0.005)	0.799(0.005)	0.946(0.005)
		0.6	0.826(0.005)	0.823(0.008)	0.995(0.008)
650	50	0.05	0.774(0.006)	0.769(0.008)	0.961(0.013)
		0.3	0.871(0.008)	0.865(0.011)	0.999(0.007)
		0.6	0.899(0.003)	0.893(0.004)	0.996(0.004)
	150	0.05	0.764(0.005)	0.762(0.008)	0.955(0.013)
		0.3	0.881(0.003)	0.837(0.004)	1.004(0.006)
		0.6	0.901(0.002)	0.896(0.003)	1.03 (0.004)
	200	0.05	0.771(0.005)	0.763(0.008)	0.957(0.009)
		0.3	0.839(0.003)	0.838(0.003)	1.01 (0.004)
		0.6	0.905(0.001)	0.897(0.003)	1.01 (0.005)
950	50	0.05	0.792(0.001)	0.778(0.001)	0.94(0.011)
		0.3	0.908(0.002)	0.907(0.003)	1.01 (0.003)
		0.6	0.933(0.002)	0.931(0.003)	1.04(0.004)
	150	0.05	0.804(0.01)	0.791(0.005)	0.955(0.015)
		0.3	0.908(0.002)	0.906(0.002)	1.01(0.01)
		0.6	0.929(0.002)	0.922(0.003)	0.995(0.005)
	200	0.05	0.796(0.003)	0.791(0.004)	0.968(0.012)
		0.3	0.908(0.002)	0.905(0.003)	0.9954(0.005)
		0.6	0.929(0.001)	0.925(0.002)	0.995(0.004)

**Table 2:** Estimated accuracy of genomic prediction (SE) and regression coefficient of true genomic breeding value on the estimated genomic breeding value (b) in the reference (A_T_) and validation (A_V_) populations at different levels of heritability, marker density, and QTL number when the positive assortative mating design was implemented.

Marker no.	QTL no.	Heritability	A_T_(SE)	A_V_(SE)	b(SE)
350	50	0.05	0.777(0.007)	0.801(0.005)	0.989(0.012)
		0.3	0.865(0.005)	0.888(0.004)	1.01(0.004)
		0.6	0.897(0.003)	0.901(0.005)	1.002(0.005)
	150	0.05	0.785(0.004)	0.803(0.007)	0.985(0.007)
		0.3	0.873(0004)	0.892(0.003)	1.01(0.006)
		0.6	0.897(0.004)	0.904(0.006)	1.01(0.006)
	200	0.05	0.783(0.006)	0.807(0.008)	0.988(0.010)
		0.3	0.873(0.004)	0.898(0.004)	1.01 (0.006)
		0.6	0.908(0.002)	0.910(0.002)	1.004(0.004)
650	50	0.05	0.822(0.004)	0.838(0.004)	0.991(0.004)
		0.3	0.921(0.002)	0.93(0.003)	1.004(0.005)
		0.6	0.948(0.002)	0.948(0.002)	1.002(0.008)
	150	0.05	0.817(0.005)	0836(0.006)	0.975(0.01)
		0.3	0.927(0.002)	0.733(0.003)	1.04(0.003)
		0.6	0.939 (0.008)	0.944(0.009)	0.996(0.004)
	200	0.05	0.839(0.010)	0.847(0.006)	0.997(0.006)
		0.3	0.927(0.002)	0.933(0.003)	1.04(0.003)
		0.6	0.939(0.001)	0.944(0.001)	1.009(0.008)
950	50	0.05	0.841(0.004)	0.849(0.006)	0.977(0.006)
		0.3	0.938(0.005)	0.948(0.001)	0.992(0.004)
		0.6	0.962(0.002)	0.966(0.001)	1.003(0.002)
	150	0.05	0.841(0.005)	0.858(0.006)	0.977(0.01)
		0.3	0940(0.002)	0.944(0.002)	1.002 (0.004)
		0.6	0.958(0.001)	0.959(0.001)	1.004(0.003)
	200	0.05	0.842(0.005)	0.862(0.004)	0.973(0.007)
		0.3	0.941(0.001)	0.946(0.002)	0.996(0.002)
		0.6	0.960(0.001)	0.964(0.001)	0.954(0.045)

**Table 3:** Estimated accuracy of genomic prediction (SE) and regression coefficient of true genomic breeding value on the estimated genomic breeding value (b) in the reference (A_T_) and validation (A_V_) populations at different levels of heritability, marker density, and QTL number when the negative assortative mating design was implemented.

Marker no.	QTL no.	Heritability	A_T_(SE)	A_V_(SE)	b(SE)
350	50	0.05	0.692(0.006)	0.680(0.011)	0.932(0.017)
		0.3	0.768(0.004)	0.759(0.007)	0.992(0.010)
		0.6	0.784(0.007)	0.765(0.005)	0.995(0.007)
	150	0.05	0.701(0.006)	0.680(0.007)	0.942(0.010)
		0.3	0.758(0.005)	0.745(0.007)	0.983(0.011)
		0.6	0.766(0.005)	0.762(0.007)	1.002(0.006)
	200	0.05	0.693(0.007)	0.701 (0.017)	0.973(0.012)
		0.3	0.756(0.004)	0.740(0.007)	0.935(0.044)
		0.6	0.708(0.045)	0.688(0.048)	0.968(0.038)
650	50	0.05	0.750(0.005)	0.702(0.004)	0.916(0.058)
		0.3	0.847(0.004)	0.846(0.005)	1.007(0.004)
		0.6	0.863(0.004)	0.861(0.004)	1.001(0.007)
	150	0.05	0.751(0.007)	0.739(0.008)	0.998(0.034)
		0.3	0.849(0.004)	0.845(0.005)	1.002(0.006)
		0.6	0.865(0.002)	0.850(0.005)	0.997(0.006)
	200	0.05	0.744(0.006)	0.747(0.007)	0.962(0.011)
		0.3	0.848(0.005)	0.848(0.004)	1.002(0.006)
		0.6	0.869(0.002)	0.858 (0.006)	0.994(0.004)
950	50	0.05	0.773(0.005)	0.767(0.006)	0.969(0.011)
		0.3	0.878(0.004)	0.875(0.004)	0.998(0.006)
		0.6	0.905(0.003)	0.884(0.006)	1.003(0.004)
	150	0.05	0.773(0.005)	0.767(0.010)	0.948(0.011)
		0.3	0.881(0.003)	0.870(0.007)	1.008(0.004)
		0.6	0.906(0.002)	0.899(0.003)	0.998(0.004)
	200	0.05	0.737(0.035)	0.769(0.006)	0.966(0.013)
		0.3	0.877(0.003)	0.870(0.004)	1.001(0.005)
		0.6	0.904(0.002)	0.893(0.002)	0.999(0.004)

**Table 4:** Estimated accuracy of genomic prediction (SE) and regression coefficient of true genomic breeding value on the estimated genomic breeding value (b) in the reference (A_T_) and validation (A_V_) populations at different levels of heritability, marker density, and QTL number when the mating design was optimized to maximize inbreeding.

Marker no.	QTL no.	Heritability	A_T_(SE)	A_V_(SE)	b(SE)
350	50	0.05	0.735 (0.007)	0.772(0.01)	0.960(0.010)
		0.3	0.825(0.003)	0.832(0.004)	1.01(0.006)
		0.6	0.837(0.005)	0838(0.009)	1.009(0.007)
	150	0.05	0.741(0.006)	0.750(0.008)	0.981(0.011)
		0.3	0.824(0.005)	0.833(0.006)	1.005(0.005)
		0.6	0.837 (0.004)	0.840(0.006)	0.994(0.009)
	200	0.05	0.757(0.005)	0.768(0.007)	0.965(0.011)
		0.3	0.817(0.005)	0.831(0.006)	1.009(0.007)
		0.6	0.823(0.001)	0.850(0.003)	1.003(0.005)
650	50	0.05	0.816(0.006)	0.813(0.007)	0.980(0.012)
		0.3	0.892(0.003)	0.891(0.005)	0.999(0.005)
		0.6	0.910(0.003)	0.908(0.006)	1.003(0.004)
	150	0.05	0.806(0.004)	0.809(0.009)	0.973(0.012)
		0.3	0.891(0.002)	0.894(0.004)	0.990(0.006)
		0.6	0.910(0.002)	0.913(0.004)	0.992(0.004)
	200	0.05	0.805(0.003)	0.807(0.007)	0.970(0.013)
		0.3	0.890(0.002)	0.890(0.003)	1.005(0.005)
		0.6	0.911(0.002)	0.916(0.002)	0.997(0.005)
950	50	0.05	0.818(0.006)	0.820(0.008)	0.979(0.008)
		0.3	0.881(0.04)	0.915(0.006)	1.01(0.009)
		0.6	0.937(0.002)	0.937(0.002)	0.995(0.003)
	150	0.05	0.813(0.007)	0.813(0.007)	0.951(0.010)
		0.3	0.916(0.001)	0.920(0.002)	1.003(0.005)
		0.6	0.938(0.001)	0.937(0.001)	1.001(0.002)
	200	0.05	0.824(0.005)	0.831(0.008)	0.985(0.009)
		0.3	0.912(0.002)	0.874(0.004)	0.999(0.005)
		0.6	0.941(0.001)	0.940(0.001)	0.996(0.003)

**Table 5:** Estimated accuracy of genomic prediction (SE) and regression coefficient of true genomic breeding value on the estimated genomic breeding value (b) in the reference (A_T_) and validation (A_V_) populations at different levels of heritability, marker density, and QTL number when the mating design was optimized to minimize inbreeding.

Marker no.	QTL no.	Heritability	A_T_(SE)	A_V_(SE)	b(SE)
350	50	0.05	0.718(0.005)	0.712(0.007)	0.967(0.011)
		0.3	0.814(0.005)	0.807(0.005)	1.004(0.007)
		0.6	0.818(0.004)	0.813(0.008)	0.990(0.008)
	150	0.05	0.721(0.006)	0.714(0.010)	0.940(0.027)
		0.3	0.814(0.006)	0.809(0.008)	0.996(0.008)
		0.6	0.813(0.003)	0.814(0.005)	1.002(0.006)
	200	0.05	0.712(0.006)	0.709(0.008)	0.969(0.010)
		0.3	0.811(0.004)	0.811(0.006)	1.45(0.004)
		0.6	0.820(0.004)	0.819(0.006)	1.002(0.006)
650	50	0.05	0.777(0.006)	0.777(0.006)	0.951(0.008)
		0.3	0.880(0.003)	0.877(0.004)	1.004(0.004)
		0.6	0.904(0.002)	0895(0.004)	1.003(0.003)
	150	0.05	0.782(0.008)	0.780(0.011)	0.964(0.011)
		0.3	0.872(0.002)	0.869(0.005)	1.004(0.006)
		0.6	0.894(0.003)	0.885(0.02)	0.997(0.005)
	200	0.05	0.743(0.038)	0.775(0.010)	0.949(0.009)
		0.3	0.837(0.003)	0.871(0.003)	0.988(0.006)
		0.6	0.898(0.003)	0.887(0.004)	0.995(0.005)
950	50	0.05	0.785(0.005)	0.758(0.003)	0.960(0.010)
		0.3	0.913(0.004)	0.902(0.004)	0.996(0.006)
		0.6	0.931(0.002)	0.928(0.002)	1.005(0.006)
	150	0.05	0.788(0.006)	0.791(0.009)	0.974(0.011)
		0.3	0.911(0.004)	0.910(0.003)	0.999(0.003)
		0.6	0.930(0.003)	0.926(0.003)	0.999(0.004)
	200	0.05	0.789(0.004)	0.784(0.008)	0.991(0.009)
		0.3	0.907(0.001)	0.903(0.002)	1.002(0.002)
		0.6	0.927(0.002)	0.920(0.002)	0.993(0.002)

### 3.2 Linkage disequilibrium

Table 6 shows the average linkage disequilibrium for various intervals between SNP pairs in each generation of the reference population when positive assortative mating design is used and there are 650 markers. Also, average linkage disequilibrium for different heritability levels, and the number of QTL in the reference population when the number of markers is equal to 650 and positive assortative mating design was implemented is presented in Table 7. Increasing the physical distance between SNP pairs decreased the LD values in all generations of the reference population (Table 6). In all generations of the reference population, the greatest values of r^2^ were observed between SNP pairs where the distance was lower than 0.1 cM (0-0.05) (Table 6). A decreasing trend of r^2^ was observed in all generations along with the increase in the distance between SNP pairs so that the lowest LDs were found for distances of 3-4 cM and 4-5 cM between SNP pairs (Table 6). Also, there were negligible differences in r^2^ values for different levels of heritability and QTL number in the reference population (Table 7).

**Table 6:** Average linkage disequilibrium (r^2^) for different intervals (BIN) between SNP pairs (N_BP_) in each generation (G) of the reference population when the number of markers is equal to 650 and positive assortative mating design was implemented.

	Generation													
	G3		G4		G5		G6		G7		G8		G9	
BIN (cM)	N_BP_	r^2^	N_BP_	r^2^	N_BP_	r^2^	N_BP_	r^2^	N_BP_	r^2^	N_BP_	r^2^	N_BP_	r^2^
(0-0.05)	194.7	0.136	196.6	0.137	193	0.138	192.9	0.138	192.6	0.140	192.4	0.222	192.4	0.141
(0.05-0.1)	194	0.088	196.1	0.089	192	0.089	192.2	0.091	191.4	0.090	191.1	0.091	182.6	0.092
(0.1-0.2)	383.9	0.057	389.5	0.118	382.2	0.060	442.3	0.059	381.3	0.059	380.8	0.059	380.6	0.060
(0.2-0.3)	386.2	0.064	390.5	0.039	383.6	0.040	383.4	0.040	382.8	0.084	382.4	0.042	382.3	0.044
(0.3-0.4)	386.2	0.069	390.9	0.031	383.4	0.070	383.6	0.033	382.9	0.033	383.8	0.034	382.2	0.035
(0.4-0.5)	386	0.128	390.7	0.026	383.1	0.027	383.1	0.028	382.4	0.028	381.9	0.029	381.8	0.060
(0.5-0.6)	386.1	0.029	390.6	0.023	383.5	0.051	383.3	0.025	383	0.025	382.3	0.026	382.1	0.027
(0.6-0.7)	385.8	0.028	390.3	0.021	383	0.022	382.8	0.022	387.3	0.023	382	0.024	381.7	0.025
(0.7-0.8)	386	0.027	395	0.019	385	0.042	384.6	0.041	383.9	0.021	383.7	0.044	383.5	0.023
(0.8-0.9)	383.9	0.016	388.6	0.018	381.1	0.039	380.7	0.019	380.1	0.020	379.8	0.021	379.6	0.021
(0.9-1)	384.7	0.027	388.8	0.017	381.7	0.018	382.2	0.018	381.1	0.019	380.7	0.020	380.9	0.021
(1-2)	3825.4	0.013	3870.6	0.013	3798.9	0.028	3795	0.015	3791.1	0.015	3787.6	0.016	3785.4	0.017
(2-3)	3776	0.023	3818	0.012	3748.9	0.011	3746.1	0.011	3740.7	0.012	3743.1	0.013	3731.6	0.013
(3-4)	3743.9	0.018	3785	0.008	3715.1	0.009	3712.3	0.010	3707.8	0.011	3704.4	0.011	3702.5	0.012
(4-5)	3703.8	0.011	3745.8	0.007	3677.8	0.008	3674.8	0.009	3669.8	0.010	3666.4	0.011	3664.1	0.011

**Table 7:** Average linkage disequilibrium (r^2^) for different levels of heritability and number of QTL in the reference population when the number of markers is equal to 650 and positive assortative mating design was implemented.

QTL number	Heritability	r^2^(SE)
	0.05	0.0169 (0.046)
50	0.3	0.0169 (0.046)
	0.6	0.0180 (0.049)
	0.05	0.0167 (0.045)
150	0.3	0.0172 (0.046)
	0.6	0.0178 (0.048)
	0.05	0.0179 (0.048)
200	0.3	0.0177 (0.045)
	0.6	0.0160 (0.043)

## 4 Discussion

The current results indicated an increase in the prediction accuracy along with an increase in the heritability level, especially in positive assortative mating design. This increase in accuracy was evident for positive assortative design compared with other mating schemes. Due to the increased genetic similarity between parents resulting from positive assortative mating, offspring may inherit stronger and more consistent genetic effects (Sunde et al., 2024). Prediction accuracy may increase due to greater genetic similarity in identifying and predicting the genetic variants associated with the trait. Positive assortative mating can contribute to the maintenance and concentration of advantageous alleles in the population by favorably mating individuals with similar phenotypes. As a result, advantageous genetic variants may become more common in offspring, facilitating their identification and use in genomic prediction models. By reducing genetic heterogeneity within the population (May et al., 2023), positive assortative mating can facilitate discovery and exploitation of the trait's underlying genetic architecture. This decline in genetic diversity may result in lower data noise, which will improve the accuracy of estimating genetic effects and ultimately improve prediction accuracy. The effects of genetic drift, which can lead to loss of beneficial alleles and a decline in genetic diversity over time, can be mitigated by positive assortative mating. Positive assortative mating can lead to more stable and accurate genomic predictions by maintaining genetic diversity and beneficial alleles in the population (May et al., 2023). Similar to the results of this study, different studies reported an increase in the genomic accuracies concurrent with the increase in the heritability level (Goddard, 2009; Howard et al., 2014; Akbarpour et al., 2021). High additive gene effects in phenotypic variation contribute significantly to the high heritability estimate, which yields high prediction accuracy. Marker effects can be estimated with lower accuracy in low-heritability traits because the correlation between the genetic and phenotypic values would be lower (Habier et al., 2011). The association between genetic markers and the desired trait is stronger when the trait has higher heritability. As a result, there may be a stronger correlation between the underlying causal variants affecting the trait and the markers. Predictive models are therefore better able to collect and apply this data to increase prediction accuracy. Higher levels of heritability also make the genetic influences on the trait more noticeable and detectable (Atefi et al., 2016). This can help reduce the level of noise in the data and make it easier to estimate the effects of genetic markers. The increase in environmental variance and the sampling variance of marker effect estimation could be the cause of the decline in genomic accuracies in low heritabilities (Akbarpour et al., 2021). Increasing the sample size might offset the drop in reference population prediction accuracy. Getting higher accuracies for traits with low heritability might be accomplished by gathering more data (Goddard & Hayes, 2007). The effect of doubling the number of phenotypic records in low-heritability traits was greater than that in high-heritability traits, but a higher number of phenotypic records is needed to reach a specific level of genomic prediction accuracy for low-heritability traits in the reference population (Hayes et al., 2009).

In the current study, as the QTL number increased from 50 to 200, the genomic accuracy showed no obvious trend or variation across all mating designs. Nevertheless, Daetwyler et al. (2010) found that genomic prediction accuracy declined as the number of QTLs increased. The LD between markers and QTLs can be increased by raising the genomic prediction accuracy and raising the marker density to 950. When compared to alternative mating schemes, the accuracy increase for positive assortative design was clearly visible. Higher marker density can improve the LD between markers and QTLs by placing more markers closer to the QTLs. This can lead to more accurate genomic prediction by increasing the resolution of the genetic map and providing more comprehensive insights into the genetic basis of the trait. Overall, more informative and reliable genetic markers for complex trait prediction can be obtained by combining high LD between markers and QTLs with higher marker density, which can improve the accuracy of genomic prediction. The more markers there are, the greater the chance of capturing more genetic variation across the genome. This can potentially make it easier to predict phenotype from genotype data and provide a more robust and thorough representation of the genetic architecture underlying complex traits. Furthermore, a larger number of markers may make it easier to identify the precise variants that have a direct impact on the desired trait. By reducing noise in the data, more markers can produce a signal that is more readable and more useful for prediction. Reducing the influence of random variations and increasing the signal-to-noise ratio can improve the accuracy of genomic predictions. Several studies reported an increase in genomic prediction accuracy due to the greater number of markers across the genome (Combs & Bernardo, 2013; Piyasatian & Dekkers, 2013; Atefi et al., 2016; Akbarpour et al., 2021). The generation interval between the two populations caused a small variation in the accuracy of the reference and validation populations. Substantial genetic differences between the two populations may exist if there is a generation gap between the validation population (tenth generation) and the reference population (third to ninth generations). Allele frequencies can vary due to selection pressures, genetic recombination, and the appearance of new mutations during the generation interval (Gregory, 2009; Buffalo & Coop, 2020). Because of these genetic changes over time, the reference and validation populations may differ in allele frequencies and genetic architecture. Consequently, these genetic differences may result in lower genomic prediction accuracy when applied to the validation population based on markers and phenotypic data from the reference population. Consequently, it is possible that the generation gap between the reference and validation populations plays a role in the variation in genomic prediction accuracy found in the study. When interpreting and applying genomic predictions to populations with different generation intervals, it is important to take these differences into account. In population genetics, the LD is a significant variable that serves as a foundation for choosing the optimal complex of genetic markers linked to causative variants in order to achieve high accuracy in MAS programs and reduce errors in the execution of breeding plans in diverse populations (Seo et al., 2018; Akbarpour et al., 2021). Due to its scale, LD uncovered marker distances and inbreeding rates, and it plays a pivotal role in genome-wide association studies (Lee et al., 2011; Akbarpour et al., 2021). The LD value would drop as the markers' distance increased. When loci on a chromosome are in close proximity, the likelihood that they will be separated by genetic recombination events is reduced. This results in alleles at these loci being more likely to be inherited together in the same combination as in the parent. Consequently, there is a higher degree of LD between loci that are physically close to each other. On the other hand, loci that are closer together on a chromosome have a higher probability of undergoing genetic recombination events that separate them. Lower LD values ​​between these loci result from the increased likelihood that alleles at these loci are inherited independently (Bailey-Wilson & Wilson, 2011). Selection, migration, mutation, genetic drift, and recombination rate are some of the factors that affect the amount of LD (Seo et al., 2018; Akbarpour et al., 2021). A population's demographic state is characterized by its effective population size, which is estimated using the LD (Lee et al., 2011; Seo et al., 2018). Since most livestock populations have relatively small effective population sizes, which result in high LD, finite population size is typically recognized as the primary cause of LD in livestock populations. The mating design affects livestock's recent effective population size and can vary significantly between breeding programs (Sun et al., 2016). A decrease in LD observed in other studies with increase in physical distance between markers is consistent with the current results (Vos et al., 2017; Barría et al., 2019; Akbarpour et al., 2021). The amount of markers or phenotypes required in the initial genome scan that uses LD to uncover QTL is clearly demonstrated by the decrease in r^2^ with distance.

## 5 Conclusion

This study investigated the influence of different mating designs on genomic prediction accuracy under various genetic architectures. The use of positive assortative mating resulted in higher genomic accuracy for reference and validation populations across all heritability, QTL number, and marker density levels. The implementation of positive assortative mating in the reference and validation populations yielded the unbiased regression coefficients of true genomic breeding value on estimated genomic breeding value. The average linkage disequilibrium values decreased along with an increase in the physical distance between SNP pairs. Positive assortative mating should be implemented among individuals in genomic evaluation programs to obtain unbiased genomic predictions with greater accuracy. This study suggests that animal breeding programs can improve offspring performance while maintaining genetic health by carefully managing mating strategies based on genetic diversity, relatedness, and inbreeding levels. To achieve optimal breeding results and ensure long-term genetic progress in animal populations, it is crucial to consider different mating designs when using genomic information. Incorporating positive assortative mating or other mating schemes into genomic evaluation programs requires careful consideration of gene interactions, environmental influences, and genetic drift to maintain the stability and effectiveness of breeding efforts. While the study provides valuable insights into the effects of mating designs on the accuracy of genomic predictions, additional research and comprehensive analyzes are essential to fully understand the effects of these factors and their complex interplay on the accuracy of genomic predictions and to develop strategies that improve breeding outcomes in animal populations. This study considers a single chromosome, simplifying the genetic architecture of the trait. Multiple genes distributed across different chromosomes actually influence traits, and the complexity of their interactions is greater than presented in the study. Because the current findings depend on specific simulation parameters, they may not be applicable to other breeding programs or species. Depending on the genetic makeup and breeding goals of different populations, positive assortative mating may or may not be beneficial in increasing the accuracy of genomic prediction.

## Funding

The authors did not receive support from any organization for the submitted work.

## Financial and non-financial interests

The authors have no relevant financial or non-financial interests to disclose.

## Availability of data

The authors affirm that all simulated data for confirming the conclusions of the current article are available from the corresponding author on reasonable request.

## Ethical approval

This article does not contain any studies with human participants or animals performed by any of the authors.

## CRediT authorship contribution statement

**Sahar Ansari:** Writing – review & editing, Formal analysis, Data curation. **Navid Ghavi Hossein-Zadeh:** Writing – review & editing, Writing – original draft, Validation, Supervision, Methodology, Investigation, Conceptualization. **Abdol Ahad Shadparvar:** Writing – review & editing, Validation, Methodology.

## Declaration of competing interest

The authors declare that they have no known competing financial interests or personal relationships that could have appeared to influence the work reported in this paper.
